# Transition to Adulthood and Adult Care: Lived Experiences of Young Adults with Perinatal HIV in the Netherlands

**DOI:** 10.1007/s10461-026-05047-z

**Published:** 2026-02-02

**Authors:** Jane N. T. Sattoe, Annouschka Weijsenfeld, Noortje van Balen, Linda van der Knaap, AnneLoes van Staa

**Affiliations:** 1 Rotterdam University of Applied Sciences, Research Centre Innovations in Care, Rotterdam, The Netherlands; 2 Wilhelmina Children’s Hospital, University Medical Center Utrecht, Utrecht, The Netherlands; 3 Academic Medical Center, Department of Internal Medicine, Division of Infectious Diseases, Amsterdam University Medical Center, University of Amsterdam, Amsterdam, The Netherlands; 4 Sophia Children’s Hospital, Erasmus University Medical Center, Rotterdam, The Netherlands

**Keywords:** Transition, Transfer, Lived experiences, Young adults, Perinatal HIV, Mental health, Peer support

## Abstract

The transition from adolescence to adulthood is a critical phase for young people living with perinatal HIV, who must navigate typical developmental milestones while managing a chronic illness and facing (fear of) societal stigma. This qualitative study explored the lived experiences of Dutch young adults (aged 20–30) with perinatal HIV, focusing on their transition to adulthood and from pediatric to adult care. Semi-structured interviews were conducted with 11 participants. While the findings are based on a small, self-selected sample and are not intended to be statistically representative, they offer in-depth insight into key challenges during transition. The findings highlight the profound impact of stigma and selective disclosure of HIV-status. Parental support was important but complex, especially when views on disclosure differed. Peer contact could provide a sense of connection, though many did not feel the need for ongoing involvement. Participants described challenges in social and professional contexts. Experiences with the transition to adult care varied, with more recent transfers being more positive. Valued key elements of transitional care included support from nurse specialists, meeting the adult provider beforehand, and a warm welcome in adult care. Despite stable medical management, the psychological burden of stigma and fear of disclosure remained significant. These findings underscore the need for tailored transition programs addressing medical, psychosocial and emotional needs, including psychological support, structured attention to family dynamics, pre-transfer meetings with adult providers, and peer support.

## Introduction

The transition from adolescence to adulthood is a critical developmental phase, marked by profound changes in identity, autonomy, and responsibility [1]. For young people living with HIV, this developmental transition often presents unique challenges, as they must navigate not only the typical milestones of emerging adulthood, but also the complexities of managing a chronic health condition within a societal context that still carries stigma and misconceptions about HIV [2–4]. Young adults with perinatal HIV report lower health-related quality of life compared to healthy age-mates [5].

Part of the transition to adulthood, is the healthcare transition from a pediatric to an adult care setting, which further complicates the challenges young people with chronic conditions face, requiring them to adjust to a different healthcare environment, establish new relationships with providers, and take greater responsibility for their care [6, 7]. Inadequate preparation for healthcare transition in young people with HIV can lead to disengagement from care and non-adherence [8]. The Dutch HIV-Monitor showed that the transition period is associated with lower levels of viral suppression and lower care retention rates in young people with perinatal HIV [9]. This was also found in a Dutch study and other international studies [10–14]. Conversely, it is known that when young people in general feel supported and confident in managing their health, they are more likely to remain engaged in care and achieve positive health outcomes [15]. Still, research mainly focuses on clinical outcomes, with limited attention to young people’s experiences.

Globally, adolescents and young adults account for a growing proportion of people living with HIV. According to UNICEF, an estimated 1.7 million adolescents (aged 10–19 years) were living with HIV in 2021 [16], with a large proportion transitioning into adulthood each year. By the end of 2023, in the Netherlands, 163 people with perinatal HIV were younger than 18 years, with 52 in the transition age range (15–17 years) [9]. These demographics underscore the importance of addressing their specific needs during a period when treatment adherence and retention in care are critical for achieving long-term viral suppression and preventing complications. Ensuring that young people are supported during this critical transition period, before and after the transfer, is essential for their health, well-being, and long-term success.

In recent years, there has been growing recognition of the need for tailored transition programs that address not only the medical, but also the psychological and social needs of young people with HIV [11, 17, 18]. Transition programs often emphasize the importance of self-management skills, such as understanding one’s medical regimen, communicating effectively with healthcare providers, and navigating healthcare systems [15]. However, the extent to which these programs address the psychosocial needs of young people with HIV is unknown. Moreover, despite the growing body of research on healthcare transitions, the voices of young people living with HIV remain underrepresented [18, 19]. Much of the existing literature focuses on clinical outcomes, such as treatment adherence and viral suppression or perspectives of healthcare professionals [20], with less attention given to the lived experiences of young people during their transition [18, 19]. Understanding the experiences of young people is key to identifying barriers and facilitators of successful transition. A successful transition could be defined as ensuring “care that is continuous, coordinated, and adapted to each youth’s development and maturity, while improving (or at least maintaining) disease control, patient satisfaction, quality of life, and social participation throughout young adulthood” [21]. Thus, a successful transition does not merely involve the transfer of medical records or appointments but requires a coordinated, purposeful process that ensures continuity of care while addressing the developmental and psychosocial needs of the individual. This study explored the lived experiences with transitioning to adulthood and adult care of Dutch young people with perinatal HIV. By centering their voices, we aim to get insight in their support needs and identify what can facilitate a successful transition.

## Methods

### Study Design

To understand the lived experiences of Dutch young adults with perinatal HIV during their transition to adulthood, we employed a qualitative approach. This approach is particularly suited to capture the subjective experiences and to explore how young people make sense of these experiences. The aim was to describe experiences as they are lived, without attempting to explain them through external theories or assumptions.

### Participants

Participants were recruited from a multicenter cross-sectional questionnaire study conducted at 15 Dutch adult HIV treatment centers [22]. Eligible participants were young adults with perinatal HIV who had transitioned from pediatric to adult care and had attended at least one visit in the adult care setting. Those who were not sufficiently proficient in reading or writing in Dutch were excluded from the study. Specialized nurses from all four Dutch pediatric HIV treatment centers identified individuals who had transferred to adult care. Eligible individuals were informed about the study and invited to participate by their HIV nurse specialist in adult care during their outpatient clinic visit. If a visit was not scheduled within three months of the study’s initiation, eligible individuals were contacted by telephone. Those who agreed to participate received an email invitation containing a link to an electronic informed consent form and the questionnaire. Participants could access the questionnaire only after signing the informed consent form.

Within the questionnaire, participants had the option to indicate their willingness to take part in an interview. Those interested provided their email addresses and were subsequently contacted by a researcher (NvB) via email. Participants who expressed interest were provided with detailed information about the study and asked to give written informed consent for the interview study. Respondents received a €20 gift voucher as compensation for their time.

### Data Collection and Privacy

Data were collected using semi-structured interviews, allowing for flexibility in the discussion while ensuring coverage of key topics. Due to the global COVID-19 crisis, interviews were conducted via Microsoft Teams in the period March to November 2021. Interviews lasted between 45 and 60 min and followed an interview guide developed based on existing literature. The interview topics covered a broad range of themes, including quality of life, autonomy, self-image, stigma, social relationships, healthcare experiences, and transition from pediatric to adult care. Sample questions included: “How do you experience your life at the moment?”, “How do you manage your condition?”, and “What are your experiences with transitioning from pediatric to adult care?”. The interviews were performed by one researcher (NvB). After the first interview, two researchers (NvB, JNTS) engaged in a reflective discussion to evaluate the interview process, identifying potential areas for improvement.

All interviews were recorded in Microsoft Teams, after which the audio was retained, and the video was deleted. The audio was used to transcribe the interviews verbatim. The audio files were securely stored on a protected cloud service, and only two researchers (NvB, JNTS) had access to the data.

### Data Analysis

Thematic analysis was conducted [23]. Transcriptions were first read and re-read to ensure familiarity with the content. The data were then analyzed inductively using an open coding process. Codes were categorized into groups, each forming a potential theme, which was subsequently reviewed to ensure consistency with both the coded fragments and the overall dataset. After that, the main themes were defined, named, and interconnected. To enhance transparency and support the themes, participant quotes were selected as illustrative examples. The first three interviews were independently analyzed by two authors (JS, NvB), with codes compared and refined through discussions. The remaining interviews were analyzed by one author (JS). Data analysis done with ATLAS.ti 23 for Windows.

### Ethical Considerations

The study was reviewed by the Medical Ethics Committee of Amsterdam UMC and local ethics committees of participating centers. It was determined that the study did not fall under the scope of the Dutch Medical Research Involving Human Subjects Act (WMO). Participants gave informed consent before the interviews, and all data were stored and handled in accordance with the General Data Protection Regulation (GDPR).

## Results

### Respondents

Seventeen young adults with perinatal HIV stated in the questionnaire that they were willing to participate in an interview. Of these, one person could not be reached, one person refrained from participation, and four did not reply to the invitation and reminders. Three females and eight males from seven different HIV treatment centers participated (*n* = 11, 65%). Participants ranged in age from 20 to 30 years. Three young adults had transitioned to adult care more than six years prior to the study, while the remaining participants had transitioned within the past six years. Of the 11 respondents, six were living with their parents and five were living independently. Four participants were still pursuing their studies; three of them held part-time jobs alongside their education, while one was not employed. Among the other seven respondents, four were employed full-time and three were not working. Respondent characteristics are presented in Table 1.

**Table 1:** Characteristics of interviewed respondents

Respondent number	Sex	Age (at the time of interview) (years)	Time since transfer to adult care (at the time of the interview) (years)
1	Male	25	> 6
2	Male	24	≤ 6
3	Male	21	≤ 6
4	Female	23	≤ 6
5	Male	21	≤ 6
6	Male	20	≤ 6
7	Female	26	> 6
8	Male	20	≤ 6
9	Male	24	≤ 6
10	Female	30	> 6
11	Male	21	≤ 6

### Themes Derived from the Interviews

Open coding resulted in 52 codes varying from healthcare related topics such as ‘medication’ and ‘transfer to adult care’ to psychosocial topics such as ‘work’, ‘relationships’, ‘stigma’ and ‘coping with negative feelings’. Results showed that stigma and disclosure were the most important themes present through all life domains of young people with HIV. Other important themes discussed were social and peer contact, professional support, HIV-medication, and transfer to adult care.

### Disclosure and Stigma

All respondents indicated that they were selective in disclosing their HIV-status, reflecting the significant role of stigma in shaping their decisions. A key factor influencing disclosure was the need to build trust before revealing their status. Participants often shared their HIV-status only with close friends or in specific situations, such as medical appointments or advocacy activities. As Respondent 7 explained, “I always found it difficult, and I still do […], when I had to go to the hospital, […] at school I would always make up an excuse of where I had to go. That was out of shame.” Young people felt sharing their status makes them vulnerable, with many respondents stating they feared the social consequences of being open about their HIV-status. Respondent 8 described this fear of being judged: “People will look differently at me.” Respondent 11 said, “It is a certain [negative] image of someone with HIV.” These fears were not unfounded, as several participants had experienced negative reactions after disclosing their status. For instance, Respondent 1 shared: “When people knew about it, I was bullied because of my HIV-status.” Such experiences often led to a reluctance to disclose again.

The level of openness thus varied widely, even within families and groups of friends. Some respondents had told their close ones about their HIV-status, while others kept it a secret even from their closest relatives due to cultural stigma. Respondent 7 said: “Actually, the biggest drawback of HIV is that you don’t necessarily feel sick, but there is still a significant stigma attached to it. In [our] culture, it is really something that is kept silent. This creates quite a lot of pressure because it is a part of who you are, yet you don’t actually share it”. Some participants indicated that their parents had advised against sharing their status, fearing social consequences. Respondent 4, for example, explained that her parents chose to tell others she had a liver disease to avoid gossip: “That is what my parents decided for me when I was a child. We lived in a small village where everyone knew everyone, and if there was gossip, everyone knew it immediately.” Similarly, respondent 10 noted that her parents had cautioned her about disclosing her HIV-status: “My parents have taught me from the beginning that some people might be shocked and not open-minded enough and have advised me not to talk about it.”

Only two respondents were in a romantic relationship at the time of the interview, but almost all had experienced sexual relations. Several respondents mentioned that they generally did not share their HIV-status with their partners, especially in casual or short-term sexual relationships. Others had shared it. An example is respondent 7: “We have been in a relationship for three years now and I disclosed it quite early”. Still, all experienced fear of their partner’s reaction: “But I have heard stories that… When people find out, they do not want to stay in contact with you anymore” (Respondent 7). Another experienced this: “When I chose to tell the person that I had HIV… That was the moment that everything went [wrong and our relationship ended] …” (Respondent 3). Some respondents never had had a long-term romantic relationship before and were unsure how they would share their HIV-status.

The work environment was another area where disclosure was carefully managed. Some participants expressed concerns that revealing their HIV-status could negatively impact their career prospects or ambitions. Respondent 2 mentioned: “It is easier for people to see you as normal if they do not know. Things generally go smoother that way.” Young people generally chose not to disclose their status in professional contexts for fear of discrimination or bias in the workplace. Most of the respondents felt that HIV did not affect their educational or professional career because of non-disclosure.

Many respondents were able to manage their condition without it significantly affecting their daily lives. Respondent 6 said: “Well, I live with it, and I feel my life is just fine. Yes, without it would be even better, but I really do not notice that compared to my friends […] that I have less, or perform less, that I am held back, because I have HIV. I do not notice that.” Respondent 9 also did not perceive any additional limitations due to his HIV-status: “Well, I just always take my pill, and I just look at it from a simple point of view. I can do what every other person can do; it does not limit me in anything, really.” Yet, the emotional toll of maintaining secrecy concerning their HIV-status persisted. Respondent 7 noted: “I think it’s mostly kind of a burden on my mental health, […] HIV and everything around it.” This burden, driven by the fear of disclosure, contributed to feelings of mental strain for several participants, although some mentioned this was more the case in their teenage years. The findings highlight how stigma plays a central role in shaping the disclosure behaviors of young adults with perinatal HIV. Respondents employed selective disclosure as a coping strategy to protect themselves from negative social reactions, particularly in the context of family, professional, and romantic relationships, and the overall narrative was one of caution, reflecting a psychological burden coming from fear of disclosure consequences.

### Social and Peer Contact

Overall, respondents describe a range of dynamics when it comes to the role of their parents in their lives, from understanding and supportive to complex and strained, indicating that while parental relationships can provide essential support, they can also be sources of conflict and emotional challenge. Respondent 6 explained: “My parents. I talk a lot with my parents especially. And also with friends sometimes. Only, friends often think the same as you, […] you are roughly on the same wavelength… And parents see things differently […].” Others described more challenging dynamics. Respondent 9 illustrated this tension: “My mother and I live very much apart, even though we live in the same house”. He further elaborated that the different perspectives and values between him and his mother created conflicts and a lack of mutual understanding. This was also experienced by other respondents who did not feel the same way about their HIV-status as their parents did, for instance due to cultural norms. Respondent 7 said: “I also think that the experiences I had with my mother, and the […] culture, have led me to make different choices than the way I was raised.”

Friendships were mostly formed during school years and through various activities or hobbies. For some respondents, friendships were a crucial support system, although only a few very close friends knew about the respondent’s HIV-status. Respondents usually bond with friends based on other similarities. Respondent 4 said: “I have a close circle of friends” and “I always have friends to fall back on.” Respondent 1 highlighted the comfort and ease found in relationships where there are mutual understandings of personal challenges, other than HIV. This was also mentioned by Respondent 2: “I have like three friends that are [close] actually and are also trying to stop the use of soft drugs [just like me]. So, we are still very close”. However, challenges, including mental and physical health issues or external issues, could impact the ability to maintain social interactions for some. Respondent 10, for instance, pointed to the stress and fatigue that social interactions can bring: “If I meet with people three times a week to catch up and be sociable, that costs me a lot of energy”.

Several respondents mentioned attending organized events and activities designed for peer contact among people with HIV in their teens. For instance, Respondent 3 was positive about outings and weekends organized by his healthcare institution. Similarly, Respondent 4 attended camps arranged by the patient organization and reported positive experiences. Such events were all labeled as “fun”. Generally, respondents reflected positively on the social interactions and the temporary sense of community fostered by these events. Yet not all respondents felt a lasting need for continuous peer contact. Despite attending events, several expressed that they did not stay in touch with others from these groups. For instance, Respondent 4 said: “Very occasionally I talk to someone, and I do follow them on social media, but not really […] every day or like, hey, how are you? But I also don’t feel the need for it anymore. […] I’m still on that Facebook group and something gets posted there. But, no, I’m not really in touch.” Respondent 11 explained that he is not focusing on HIV: “I consciously chose [not to engage with peers], not much need for it, I guess. No, I just live my life without really thinking about the disease very much. So, I don’t have a very big need for [contact with peers].” While organized peer contact thus provided some with a sense of community and shared experience during adolescence, many respondents did not pursue long-term or deeper connections with peers because they did not feel the need for this.

### Professional Support

When asked about receiving professional support to prepare for the transition to adulthood including the transfer to adult care, young people mostly did not recall receiving additional support, but they also did not really feel the need for extra support from their regular healthcare team. Respondent 5 mentioned that nurses and nurse specialists offered a more personal and in-depth approach to explaining HIV and related issues (such as use of alcohol and drugs) compared to doctors. He said: “The pediatric nurse specialist did go into a bit more depth about what HIV meant, than the doctor I had. She had, like, booklets with pictures in them that explaining HIV and things like that. She also explained what drugs and alcohol can do to HIV […]. So, I went deeper into it with her”. Respondent 1 mentioned getting support from a “care coordinator”, while respondent 2 said: “I never really thought about it to be honest. […] I never thought about getting professional help. I’m the kind of person… I do a lot on my own. You know, if I face a difficulty, I just pick myself up and try to like figure it out.” Still, several respondents mentioned the need for more structured mental healthcare for young adults with HIV. Respondent 7 suspected a relationship between HIV and mental health issues, especially among young people, but acknowledged a lack of empirical evidence to support this feeling. She added: “I think this is an important issue for young people, at least until the age of 30 years.” Yet, overall, the mental healthcare provided to young adults was often regarded as too voluntary or optional by respondents. Respondent 10 explained: “The mental care is a little too optional. I think that every young person who has to deal with HIV, they just need help with that and it’s a little too optional, because I remember once […] I was asked whether I needed psychological help, and [… this] was asked that in front of my parents and they were always like ‘no, we don’t need that’. So that’s what I said. And then […] it was never asked again, at least in my experience.”

### HIV-Medication

Most respondents did not feel HIV had a big impact on their daily lives apart from that they have to take their medication, often only once a day. Attitudes towards taking HIV-medication differed. Some respondents showed rigorous adherence to their medication schedules. For example, Respondent 2 mentioned: “I have an alarm at 6 p.m., one at 7 p.m., and one at 8 p.m., that is my time range to take my medication”. Respondent 3 stated, “I think I miss it three times a year or so. Basically, [I take the medication] every day”. However, other respondents mentioned that they did not always take their medication. Some reasons for this include forgetfulness due to a “busy day” or simply being “too tired” to remember to take their medication (Respondent 2). Respondent 7 mentioned that she used to forget to take her medication, because she was conscious of hiding her medication from others, but she also mentioned this has improved over time. A common strategy among the respondents is the use of alarms and reminders to ensure medication is taken on time. Respondent 4 said: “At 8 p.m. my alarm goes off on my phone. Everyone in my direct contacts knows that I must take my medication then.” Related to HIV-medication, respondents mentioned the inconvenience of frequent pharmacy visits. Respondent 8 indicated: “I would like it to be once a year instead of every three months”.

### Transfer to Adult Care

The experiences with the transfer from pediatric care to adult care varied widely between respondents. Some young adults found it smooth and well-organized, while others perceived it as abrupt and unpleasant. The respondents who experienced a lack of communication and guidance were the ones that transferred more than six years before the date of the interview. Respondent 1 was simply informed about the next appointment being in adult care without any further guidance. He said: “Communication from the children’s hospital: there are shortcomings there. At least back then. We are quite a few years further now, though. So, I don’t know about now. But also, the transfer from the children’s hospital to the adult hospital. I literally didn’t have that. Just zero.”

Of those who felt prepared and supported, an important reason was that they were introduced to their new healthcare provider(s) before transfer to adult care. These were all respondents who transferred in the past six years. Respondent 2: “I was kind of ready for it because [my pediatrician] showed me several times who [the adult doctor] was. She explained me the difference. She explained me that I had to go after becoming 18 years old […] and showed the person who was going to take care of my file. She [introduced] him to me and we kind of socialized. And afterwards, when the transfer was done, my next appointment was directly with him. So, it wasn’t complicated for me to go to adult [care].” Respondent 5 also shared his positive experience and mentioned that he had a gradual transition to adult care: “My doctor and nurse, […] they first explained that I would have to transfer to adult [care]. So, every appointment after that, the adult doctors were there. Who would then explain what the transition will be like. […] And then they would also show me the adult clinic. So, it was actually a gradual transition, it went smoothly, and I wasn’t bothered by anything.” Respondents who did not meet their new adult care doctor before the transfer, thought it would be a good idea to have had that chance. Respondent 9 said: “[…] you would only meet him on your own the moment you would see him [in adult care] and that makes it much more stressful for someone, because […] you would like to see him once before you actually go and meet him [in adult care], because then you can still indicate ‘I don’t like him’, or ‘I don’t like him this and this’, but you don’t [get] that chance. You just must go and that’s that.”

All respondents looked back at pediatric care with positive feelings, while they expressed mixed feelings about the adult care department or hospital. One respondent noted that he appreciated the sense of community and resources available to adults with HIV, which made him feel less alone (Respondent 2). However, Respondent 9 said that although she initially wanted to move to the adult department inspired by an older friend, she later found the adult care to be a cold and impersonal in contrast to the more personal care in the pediatric department: “And that’s what you do get at the children’s clinic, […] they ask ‘hey, how’s school going, did you make friends’, you know,… If there had been a youth clinic that could kind of mix the aspects of the children’s clinic and the adult clinic a little bit, that you could have several young people, several people from 18 to 23 together and could make a kind of community out of that as well, and I think it would also make it a bit more attractive for young people to go to the doctor, because I know a lot of people who don’t like going to the doctor, purely because it’s so businesslike”.

## Discussion

### General Discussion

This study showed that, while the medical consequences of HIV do not greatly affect the daily lives of young adults with perinatal HIV in the Netherlands, stigma and disclosure are major issues shaping how they deal with their condition in daily life. By focusing on the Netherlands, this study contributes a European perspective to a literature on perinatally acquired HIV transition that is largely dominated by studies from the United States and sub-Saharan Africa.

The findings highlight that (fear of) stigma plays a central role in forming disclosure behaviors. Young people are often very selective in disclosing their HIV-status to protect themselves from negative social reactions. They fear the consequences of disclosure for relationships, career and future ambitions, which poses a significant mental burden on them. Furthermore, they shared the need for more structured mental healthcare. Across the literature, there is growing recognition of the complex relationship between HIV-disclosure and mental health among young people with perinatal HIV [24, 25]. Compared to those infected later in life, adolescents with perinatal HIV often experience early and ongoing exposure to the virus and treatment, along with cognitive difficulties and psychosocial stressors like parental loss and stigma [19, 26]. A key difference is that they typically learn about their HIV-status during childhood or early adolescence, which can have lasting emotional and psychological effects [24]. Studies consistently highlight a high prevalence of mental health challenges in this population, including depression, anxiety, suicidal ideation, and emotional distress [24, 25, 27]. Delayed or poorly supported disclosure of HIV-status is linked to increased psychological distress [24, 28]. It is specifically recommended that mental health of young people should be addressed throughout the transition to adulthood and adult care [25]. Despite this acknowledgement, mental healthcare is rarely offered and underutilized leading to mental health problems being underdiagnosed in this group [24, 27, 29]. While some evidence-based interventions show promise, there is a pressing need for integrated care that addresses both HIV and mental health needs, and that is tailored to support young people through the disclosure process and beyond [28].

As young people transition into adulthood, they describe drawing on various sources of support, with parents and caregivers playing a particularly significant, but complex role. The results showed that while parental involvement can be a key source of emotional and practical support, it can also be a source of tension, especially when there are disagreements around disclosure strategies. Disclosure and stigma have been shown to strain family relationships, as highlighted in research on treatment challenges and secondary prevention among adolescents and young adults with early-acquired HIV [14]. A review on the mental health of youth living with HIV notes that the shift in care responsibility from parent to adolescent is often stressful [25]. Several studies emphasize that parental support is critical during this transition, with its absence identified as a barrier to successful transition [10, 18, 19, 30, 31]. Conversely, strong family support is frequently cited as a facilitator of successful transition. Multiple studies advocate for incorporating family involvement as an essential element of transitional care planning [10, 18]. However, the support needs of parents of children with chronic conditions themselves are often overlooked, and very few transitional care interventions are designed to include or assist them [32]. Since parents are an important source of support for young people, greater attention to their own support needs during this critical period is needed.

In the literature, peer support is identified as another potential source of support during the transition to adulthood. A qualitative study exploring social outcomes among young adults with perinatal HIV for instance, emphasized a clear need for peer support, particularly following the transition to adult care [33]. Participants in our study generally responded positively to experiences with peer contact in the past, appreciating the sense of belonging these events provided. However, many did not express specific benefits or a desire for continued peer engagement, and did not maintain contact beyond the events. This contrasts with existing literature, which often highlights peer support as a valuable and effective approach to delivering psychosocial and self-management support for young people with chronic conditions [34]. One possible explanation for this difference in findings may lie in the type of peer support offered. Participants in this study often referred to temporary or event-based programs, such as camps, while peer support can vary widely in format, content, structure, duration, and intensity [34]. More structured, ongoing peer support initiatives like peer-buddy systems may be better suited to the needs of this population. Another explanation may be that that the participants in this study, who were engaged in care and adherent to treatment, represent a group that feels less need for peer-based support due to their relatively stable circumstances.

Experiences with the transition from pediatric to adult care varied considerably among respondents. While some described the process as smooth and well-organized, others experienced it as abrupt and lacking in support. Those who reported poor communication and limited guidance had typically transferred more than six years before the interview, suggesting that transitional care for young people with HIV in the Netherlands has improved in recent years. The support young people received from a care coordinator or nurse specialist was highly appreciated. Nurse specialists often take on the role of care or transition coordinators and are widely recognized for their crucial role in delivering high-quality transitional care [15, 35–37]. A smooth and supportive introduction to adult care was seen as essential for maintaining engagement, with participants emphasizing the importance of meeting their new healthcare providers in advance. This highlights the value of structured transition clinics and warm handover processes. These findings align with broader transition literature, which shows that key elements of successful transitional care, such as early engagement, feeling acknowledged by the adult care team, and building trusting relationships, are consistent across hiv-populations and different chronic conditions [20, 38–40]. These insights reinforce the need for tailored, person-centered transition programs that ensure continuity of care and foster long-term engagement. Our findings align with broader transition frameworks, including the Six Core Elements of Got Transition^®^ [41] and the On Your Own Feet framework [42], in emphasizing the importance of developmentally appropriate, coordinated, and person-centered support during transition.

### Strengths and Limitations

The study included adolescents from multiple HIV treatment centers across the Netherlands, encompassing both those who transitioned to adult care in earlier years and those who did so more recently. This allowed for the observation of improvements in transition experiences over time. Another strength of this study is that data collection continued until data saturation was achieved, meaning when analysis of consecutive interviews yielded no new themes or substantive insights. This increases the credibility of the findings and suggests that the experiences of participants were captured in sufficient depth. Another strength is the attention to ethical data handling. All audio files were securely stored on a protected cloud service, access was limited to two researchers, and transcriptions were pseudonymized to protect participants’ privacy. Additionally, researcher reflexivity was built into the process. After the first interview, two researchers engaged in a reflective discussion to evaluate and improve the interview process, which contributed to the overall quality and consistency of data-analysis. All authors actively participated in the final interpretation of findings. Also, the interviewer was not previously involved in the HIV field and was not a healthcare professional, which may have supported participants in speaking freely about their experiences outside a clinical context.

On the other hand, the participants in this study represent a self-selected group who are currently engaged in care and adherent to treatment. Also due to the small sample size, demographic variables were not used as analytic categories but were considered where relevant in interpreting individual accounts. This may limit the extent to which the findings capture the full spectrum of experiences and challenges in young people with perinatal HIV related to transition. As a result, the perspectives of those who have disengaged from care, or face more significant barriers may be underrepresented and the differences between sex and cultures remain unexplored. Nevertheless, by centering the voices of those who participated, the study still offers meaningful insights into their support needs and highlights key factors that facilitate a successful transition.

Another limitation of this study is that all interviews were conducted via Microsoft Teams due to COVID-19 restrictions at the time of data collection. This may have influenced the quality of the conversations. Building rapport and picking up on non-verbal cues can be more challenging in an online setting, potentially affecting participants’ comfort in sharing personal or sensitive experiences. At the same time, conducting interviews online also had potential benefits, including increased flexibility, reduced travel burden, and possibly a greater sense of safety and comfort for participants when discussing sensitive topics.

## Conclusion

This study aimed to gain insight into the support needs of young people with perinatal HIV and what can facilitate a successful transition to adulthood and adult care. Although all participants were still engaged in care and claimed to be adherent to treatment, stigma, disclosure, and (consequently) mental health stood out as key challenges that shaped their experiences. Parental support was seen as important but also complex, especially when there were differing views on disclosure. Peer contact was appreciated for providing a sense of connection, though many did not feel the need for ongoing involvement. Experiences with the transfer to adult care varied, but the group that transferred more recently had more positive experiences. Key elements of transitional care that were valued by young people were: support from nurse specialists, meeting the adult healthcare provider before transfer and receiving a warm welcome in adult care. Young people expressed a need for more psychosocial support during transition. Although medical management was generally stable, stigma and its impact on mental health emerged as central, cross-cutting issues shaping experiences across social, relational, and healthcare domains during transition. Given that disengagement from care often makes young people difficult to reach for both support and research, future efforts should prioritize preventive psychosocial support during the transition period. Transition programs benefit from systematically offering psychological support, addressing family dynamics in a structured manner, ensuring pre-transfer meetings with adult providers, and providing more structured forms of peer support.

## Data Availability

Due to privacy concerns, the data generated and analyzed during the current study are not publicly available.
